# 

**Published:** 1873-06

**Authors:** 


*List of Cash Payments to the Record.*

Dr. F. McCrimmon, Georgia, $2 50: Dr. C. B. Slaughter, Mississippi, 2 50; Dr. Oliver O. Wozencraft, Arkansas, 2 50; Dr. Benjamin Rhett, South Carolina, 2 00; Dr. J. C. Kirkman, North Carolina, 2 50; Dr. P. D. Laster, North Carolina, 2 50; Dr. J. R. Allen, Tennessee, 2 50; Drs. Cox & Ellis, Mississippi, 2 50;

Dr. K. B. Murchison, Georgia, 2 50; Dr. G. W. Thomas, Georgia, 2 50; Dr. S. E. Winnemore, Alabama, fifty cents balance; Dr. James Ray, Louisiana, 2 50; Dr. Joseph W. Day, Louisiana, 2 50; Dr. W. P. Lawton, Arkansas, 2 50; Dr. P. Taylor, Mississippi, 2 50; Dr. J. R. Hicks, Mississippi, 2 00; Dr. J. L. Hill, Oregon, 2 50; Dr. A. C. Halbert, Mississippi, 2 50; Dr. J. P. Cheney, Georgia, fifty cents, balance; Drs. Pool & Sumrall, Mississippi, 2 50.

### The Southern Medical Record.

EDITED BY

T. S. POWELL, M.D.,

AND

W. T. GOLDSMITH, M.D.

### ASSOCIATE EDITORS:

GEORGIA.

Robert Battey, M.D.
R. C. Word, M.D.
W. L. Kirkpatrick, M.D.
James A. Long, M.D.
W. L. M. Harris, M.D.
H. L Battle, M.D.
W. C. Musgrove, M D
L B. Bouchelle, M.D.
John C. Drake, M.D.
E. F. Knott, M.D.
Thomas Burdell, M.D.
E. H. W. Hunter, M.D.
V. S. Hitt, M.D.
J. E. Pope, M.D.
J. A. Hunnicutt, M.D.
A. H. Brantley, M.D.
J. J. Knott, M.D.
J. C. Wisdom, M.D.
W. W. Leake, M.D.

ALABAMA.

J. C. Knox, M.D.
Geo. F. Taylor, M.D.
J. B. Barnett, M.D.
S. M. Hogan, M.D.
D. S. Hopping, M.D.
J. T. Searcy, M.D.
S. F. Hill, M.D.

MISSISSIPPI.

C. B. Gallaway, M.D.
Edward Lea, M.D.
S. V. D. Hill, M.D.
W. B. Harvey, M.D.
J. W. M. Shattuck, M.D.
E. T. Henry, M.D.
T. P. Lockwood, M.D.
Robert Kells, M.D.

VIRGINIA.

Charles S. Lewis, M.D.
John H. Claiborne, M.D.
F. Horner, Jr., M.D.
R. J. Preston, M.D.
A. S. Payne, M.D.
J. E. Lockridge, M.D.
J. S. Wharton, M.D.
Wm. O. Owen, M.D.
Sam'l P. Ripley, M.D.
Benj. Blackford, M.D.
E. A. Drewry, M.D.
Rob B. Tunstall, M.D.
A. J. Terrell, M.D.
W. F. Barr, M.D.
L. B. Anderson, M.D.
S. L. Jepson, M.D., W. Va.
J. M. Lazzell, M.D.

SOUTH CAROLINA.

E. T. McSwain, M.D.
G. M. Rivers, M.D.

TENNESSEE.

J. D. Tucker, M.D.
Swan M. Burnett, M.D.

TEXAS.

S. M. Welch, M.D.
T. J. Heard, M.D.
W. A. Heard, M.D.
A. R. Kilpatrick, M.D.

ARKANSAS.

A. D. Hodge, M.D.
J. K. Hodge, M.D.

NORTH CAROLINA.

J. R. Hughes, M.D.
S. S. Satchwell, M.D.
A. B. Pierce, M.D.
H. Kelly, M.D.
Geo. A. Foote, M.D.
E. A. Anderson, M.D.
H. T. Bahnson, M.D.
Willis Alston, M.D.
Thos. F. Wood, M.D.
N. J. Pittman, M.D.

### CORRESPONDING EDITORS:

J. M. Toner, M.D., Washington City, D. C.
John H. Weir, M.D., Illinois.
Thomas J. Gallaher, M. D, Pennsylvania.
Ely Van DeWarker, M. D., New York.
Robert Robson, M.D., Indiana.
C. C. F. Gay, M.D., New York,
*Surgeon of Buffalo General Hospital.*
W. T. Taylor, M.D, Kentucky.
J. Orne Green, Boston, Massachusets.
B. W. Stone, M.D., Kentucky,
*Assistant Physician Western Lunatic Asylum.*
James Tyson, M.D., Philadelphia, Pa.
W. W. Leen, M.D., Philadelphia.

M. H. Henry, M.D., New York,
*Surgeon to New York Dispensary, Department of Venerial and Skin Diseases.*
Wm. R. Whitehead, M.D., Colorado Territory.
A. Patton, M.D., Indiana.
Benjamin Howard, M.D., New York.
Chas Kearns, M.D., Kentucky.
S. C. Busey, M.D., Washington, D. C.
A. W. Hobson, M.D., Arkansas.
A. W. Calhoun, M.D., now of Berlin, Prussia.
T. Curtis Smith, Ohio.
Geo. Strawbridge, M.D., Philadelphia.

☞ *All Business Letters should be addressed to POWELL & GOLDSMITH, Post Office Box 527, Atlanta, Georgia.*

